# Elimination of *cis*-cleavage in CRISPR diagnostics for one-pot rapid nucleic acid detection

**DOI:** 10.1093/nar/gkag267

**Published:** 2026-03-26

**Authors:** Wenhao Yin, Zhili Jin, Qingyuan Jiang, Shuqi Jin, Xinping Wang, Ruyi He, Bin Qiao, Jie Qiao, Xianhua Zhang, Yi Liu

**Affiliations:** State Key Laboratory of Biocatalysis and Enzyme Engineering, School of Life Sciences, Hubei University, Hubei 430042, China; School of Life Science and Technology, Wuhan Polytechnic University, Hubei 430023, China; Department of Cardiology, Zhongnan Hospital of Wuhan University, Hubei 430071, China; State Key Laboratory of Biocatalysis and Enzyme Engineering, School of Life Sciences, Hubei University, Hubei 430042, China; State Key Laboratory of Biocatalysis and Enzyme Engineering, School of Life Sciences, Hubei University, Hubei 430042, China; State Key Laboratory of Biocatalysis and Enzyme Engineering, School of Life Sciences, Hubei University, Hubei 430042, China; School of Life Science and Technology, Wuhan Polytechnic University, Hubei 430023, China; Department of Oral and Maxillofacial Surgery, The First Affiliated Hospital of Zhengzhou University, Zhengzhou University, Zhengzhou 450001, China; School of Life Science and Technology, Wuhan Polytechnic University, Hubei 430023, China; State Key Laboratory of Biocatalysis and Enzyme Engineering, School of Life Sciences, Hubei University, Hubei 430042, China; State Key Laboratory of Biocatalysis and Enzyme Engineering, School of Life Sciences, Hubei University, Hubei 430042, China; BravoVax Co., Ltd., Wuhan, Hubei 430075, China

## Abstract

Current one-pot clustered regularly interspaced short palindromic repeats diagnostics are limited by the *cis*-cleavage activity of Cas nucleases, which leads to amplicon degradation during amplification. Here, we report a streamlined strategy that overcomes this limitation. By integrating a bipartite split-crRNA into Cas12a (SCas12a), we separate target recognition from PAM dependency and completely eliminate *cis*-cleavage while preserving robust *trans*-cleavage. This strategy is broadly applicable for one-pot testing, compatible with recombinase polymerase amplification, RT–RPA, and loop-mediated isothermal amplification, as well as multiple Cas12a orthologs, including As, Lb, and Ct Cas12a. Moreover, the SCas12a accelerates one-pot testing with 100–1000-fold improved sensitivity and achieves >10-fold reduction in time-to-signal, enabling detection of targets at attomolar levels within 30 min. Additionally, it provides single-base resolution with up to 91-fold selectivity. The system has been successfully applied to detect HPV16, SARS-CoV-2, and TP53 SNPs in clinical samples. Together, we have developed a PAM-independent and *cis*-cleavage-free one-pot Cas12a assay, which holds strong potential for point-of-care diagnostics.

## Graphical Abstract

**Graphical Abstract:**
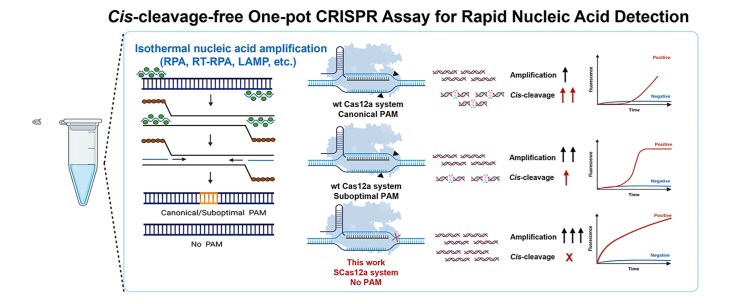

## Introduction

Nucleic acid diagnostics are essential for controlling infectious diseases, genetic screening, and environmental monitoring. However, an ideal diagnostic platform must combine high sensitivity and specificity with speed, simplicity, and low infrastructure needs [1, 2]. Although accurate, gold-standard methods like polymerase chain reaction (PCR) and sequencing are limited to centralized labs [3–5], a limitation that became especially evident during the SARS-Cov-2 pandemic. Isothermal nucleic acid amplification (INA), such as recombinase polymerase amplification (RPA) [6] and loop-mediated isothermal amplification (LAMP) [7], offers a promising alternative by eliminating the need for thermocycling, thereby enabling cost-effective and portable diagnostic assays. However, sole isothermal amplification assays are often subject to nonspecific amplification, resulting in false-positive results [8, 9].

Over the past decade, clustered regularly interspaced short palindromic repeats (CRISPR) technology, initially harnessed for gene editing, has expanded into molecular diagnostics, driven by the discovery of *trans*-cleavage activities in effector proteins such as Cas12 [10], Cas13 [11], Cas12a2 [12], and catalytically active Cas9 [13]. Among Cas enzymes, Cas12a is the most widely used due to its ability to specifically detect target nucleic acids and then nonspecifically cleave nearby single-stranded DNA (ssDNA). Combining CRISPR/Cas systems with INA allows for highly sensitive nucleic acid detection. However, early CRISPR-based diagnostic methods such as DETECTR [10], HOLMES [14], and SHERLOCK [11] involve a two-step procedure: nucleic acid amplification followed by the addition of CRISPR reagents. This approach increases procedural complexity, prolongs testing time, and heightens the risk of contamination due to nucleic acid aerosols.

Recently, several one-pot INA/Cas12a assays have been developed, enabling simultaneous and highly efficient amplification and CRISPR-mediated detection within a single reaction tube [15–25]. However, a major challenge remains in this field: the *cis*-cleavage activity of Cas12a can severely interfere with isothermal amplification. Multiple strategies have been investigated to overcome this issue. For example, some studies have employed spatial segregation of reagents [26–28], such as glycerol or sucrose, to create phase separation between amplification and detection solutions. Additionally, light-responsive crRNA strategies have been innovatively applied to control (i.e. activate or inhibit) Cas12a cleavage activity [29–33]. Furthermore, targeting suboptimal PAM sequences with LbCas12a-crRNA can reduce its *cis*-cleavage activity [34], thereby improving one-pot detection compared to canonical PAM sequences. This method is promising due to its simplicity and speed, although the limitation of PAM sequence compatibility still needs to be addressed. In addition, Cheng *et al.* [35] showed that sodium heparin can precisely regulate Cas12a’s *cis*-cleavage activity by disrupting Cas12a-crRNA binding, allowing the accumulation of isothermal amplification products and generating strong fluorescence signals. Despite these advances, existing one-pot detection systems still face limitations in terms of prolonged detection times and operational complexity. Therefore, there is an urgent need to develop a universally applicable one-pot detection method that is both PAM-independent and free from *cis*-cleavage interference.

Our group recently developed a Cas12a-based assay named SCas12a [36, 37], which integrates Cas12a with a split crRNA to enable direct RNA detection. In this study, we re-evaluated this system and unexpectedly found that it maintains robust *trans*-cleavage activity on double-stranded DNA (dsDNA), even in the absence of canonical or reported suboptimal PAM sequences. In addition, the SCas12a system exhibits negligible *cis*-cleavage activity upon recognizing dsDNA targets. Based on these critical findings, we established a universal one-step CRISPR detection method in which isothermal amplification and Cas12a-mediated detection occur simultaneously within a single reaction tube. Compared to the conventional one-pot CRISPR diagnostics, this method accelerates the reaction speed by at least tenfold and significantly improves both sensitivity and reliability. Moreover, by utilizing a thermally stable Cas12a ortholog from *Clostridium thermobutyricum* (CtCas12a), the method can be seamlessly integrated with LAMP at 55°C, enabling one-pot detection of clinical human papillomavirus 16 (HPV16) within 20 min. Additionally, it can be coupled with RT–RPA for highly sensitive detection of RNA targets, such as SARS-CoV-2 RNA from nasal swab specimens. Furthermore, the SCas12a assay exhibits remarkable specificity for dsDNA point mutations and serves as a robust tool for single-nucleotide variant (SNV) detection of human TP53 mutations in tissue samples from patients with oral cancer.

Together, we have established a straightforward strategy for constructing Cas12a systems that are both PAM- and *cis*-cleavage-independent, thereby unlocking the full potential of one-pot CRISPR-based diagnostics and offering significant advantages over other widely used tools for rapid molecular detection.

## Materials and methods

### Ethical statement

In this study, human nasal swab specimens for SARS-CoV-2 detection were collected and provided by the College of Medicine and Health Science under a protocol approved by the ethics committee at Wuhan Polytechnic University. Human vaginal swab samples for HPV16 detection were obtained from the pharmacy department at Henan Cancer Hospital, following an approved ethics protocol from Zhengzhou University. Additionally, human oral cancer tissue samples were collected from cancer patients and healthy controls at the First Affiliated Hospital of Zhengzhou University, under another ethics committee-approved protocol. All participants provided written informed consent.

### Materials

FAM-labeled ssDNA probes, DNA and RNA oligonucleotides, and gene fragments were synthesized by Sangon Biotech (Shanghai, China). The sequences of all the nucleic acids are listed in Supplementary Table S1. Buffer preparation reagents were purchased from Sinopharm Chemical Reagent Co., Ltd. (Beijing, China). NEB Buffer 2.1 was supplied by New England Biolabs (Beijing, China). The RNA extraction miRCURY LNA RT Kit was obtained from Qiagen. RT–RPA and RPA kits were provided by Weifang Amp-Future Biotech (Changzhou, China), and the LAMP kit was sourced from New England Biolabs (Beijing, China). Proteinase K (Takara, Dalian, China) was used for protein digestion. Ultrapure water was used throughout all experiments, and all DNA/RNA samples were dissolved in DEPC-treated water and stored at −20°C.

### Protein expression and purification

The gene fragments of AsCas12a, LbCas12a, and CtCas12a were cloned into the 6×His-pET-28a(+) vector and used to transform *Escherichia coli* BL21 (DE3). When the culture reached an OD_600_ of 0.8 at 37°C, protein expression was induced by adding 0.5 mM isopropyl β-D-1-thiogalactoside, followed by incubation at 18°C for 16 h. Cells were then harvested by centrifugation. The target proteins were purified from the cell lysate using Ni-NTA resin and eluted with a buffer containing 20 mM Tris–HCl, 500 mM NaCl, and 500 mM imidazole. The eluted protein was further purified by gel filtration (Superdex 200 Increase 10/300 GL) in a buffer containing 20 mM Tris–HCl and 200 mM NaCl (pH 7.5). The final storage buffer consisted of 20 mM Tris–HCl (pH 7.5), 250 mM NaCl, and 5% glycerol. Purified proteins were concentrated using a 100 kDa MWCO filter, quantified by Bradford assay, flash-frozen in liquid nitrogen, and stored at −80°C.

### Gel electrophoresis analysis of *cis-*cleavage activity

Initially, WT Cas12a and SCas12a RNPs were reconstituted in reaction buffer by combining each Cas12a nuclease with its corresponding full-length or split crRNA at a 1:2 molar ratio, followed by a 20-min incubation at room temperature. A target dsDNA plasmid, P-HPV16, was constructed by inserting a 20-bp HPV16 DNA fragment containing a PAM sequence and another 20-bp fragment lacking a PAM sequence into the pcDNA3.1(+) vector, yielding a plasmid of 5430 bp in total length. The cleavage reaction was carried out in a 10 μl mixture containing 1 μl of 10× NEB Buffer 2.1, 100 nM Cas12a, 200 nM crRNA (or 200 nM split RNA), and 10 nM HPV16 plasmid. The reaction was incubated at 37°C for 30 min and then terminated by heating at 90°C for 10 min. Cleaved DNA fragments were analyzed by 2% agarose gel electrophoresis. The gel was then stained with GoldView and visualized under a UV transilluminator for image capture.

### qPCR analysis of *cis-*cleavage activity

First, wild type (WT) Cas12a and SCas12a were reconstituted by incubating each nuclease with its cognate full-length or split crRNA at a 1:2 molar ratio in reaction buffer for 20 min at room temperature. Three HPV16-derived dsDNA substrates were generated by qPCR: one containing a canonical PAM (TTTG), one with a suboptimal PAM (TTTA), and one lacking any PAM (PAM-free). These dsDNAs served as cleavage substrates in subsequent assays. The qPCR reaction mixture was then combined with the Cas12a cleavage system—comprising 1 μl of 10× NEB Buffer 2.1, 100 nM Cas12a RNP, 200 nM crRNA (or split crRNA), and 10 pM dsDNA substrate—and analyzed in real time on a CFX96 Touch system (Bio-Rad, CA, USA). Control reactions contained identical dsDNA concentrations but omitted Cas12a RNP.

### Fluorescence detection of trans-cleavage activity

The cleavage reactions were carried out in a final volume of 10 μl containing 1 μl of 10× NEB Buffer 2.1, 250 nM Cas12a enzyme, 500 nM crRNA (or 500 nM split RNA), 10 nM dsDNA with varying PAM sequences, and 1000 nM fluorescent probes. The reactions were monitored at 37°C for AsCas12a and LbCas12a, and at 55°C for CtCas12a using a CFX96 Touch real-time PCR system (Bio-Rad, CA, USA).

### RPA and RT–RPA

One lyophilized RPA pellet was resuspended in 29.4 μl of buffer A, 14.1 μl of nuclease-free water, 2 μl of 10 μM RPA forward primer, and 2 μl of 10 μM RPA reverse primer to prepare the RPA reaction mix, following the manufacturer’s instructions (Weifang Amp-Future Biotech). For the RT–RPA reaction, 0.9 μL of RNase H (50 U/μL stock; New England Biolabs, NEB) and 0.45 μL of SuperScript IV reverse transcriptase (Thermo Fisher) were added to the RPA mixture. The reaction was carried out at 37°C.

### RPA- and RT–RPA-mediated one-pot testing using AsCas12a

One-pot reactions were carried out in 20 μl reaction volumes containing 100 nM AsCas12a RNP, 500 nM FQ ssDNA reporter, dsDNA substrate, and RPA or RT–RPA components. The RNP complex, FQ ssDNA reporter (6 μl), and RPA mixture (10 μl) were added to each reaction well, followed by the addition of 2 μl of reaction buffer and dsDNA activator. Reactions were then monitored using a CFX96 Touch Real-Time PCR System (Bio-Rad, CA, USA) at 37°C.

### Gel electrophoresis analysis of the amplicons in the one-pot RPA assay

The one-pot reaction (20 μl total) contained 100 nM AsCas12a RNP, 1 pM dsDNA substrate, and RPA components. Ten microliters of pre-assembled RNP–RPA mixture was added to each well, followed by reaction buffer and 2 μl of dsDNA activator. Reactions were incubated at 37°C for 50 min. At designated time points, reactions were heat-inactivated at 95°C and treated with proteinase K; products were then resolved on a 2% TAE agarose gel. Gels were stained with GoldView and imaged under UV transillumination.

### LAMP-mediated one-pot testing using CtCas12a

The LAMP/SCas12a one-pot testing was conducted in a 25 μl reaction volume with 100 nM CtCas12a RNP, 500 nM FQ ssDNA reporter, 1.4 mM of each dNTP, 8 mM MgSO_4_, 50 mM taurine, 0.32 U/μl Bst 2.0 WarmStart DNA polymerase, 1 × primer (0.2 μM F3/B3 primers, 0.4 μM Loop F/Loop B primers, and 1.6 μM FIP/BIP primers). Additionally, 1 × reaction buffer composed of 20 mM Tris–HCl, 10 mM (NH4)_2_SO_4_, 2 mM MgSO_4_, and 0.1% Tween 20 was included, along with 2 μl of substrates. Fluorescence values were monitored using a CFX96 Touch Real-Time PCR System (Bio-Rad, CA, USA) at 55°C for 60 min.

### qPCR and RT-qPCR assay

For the detection of specific DNA targets, primers and a qPCR detection kit were obtained from GenePharma (Suzhou, China). Clinical samples (e.g. HPV16) were analyzed according to the manufacturer’s protocol using a CFX96 Touch Real-Time PCR System (Bio-Rad, CA, USA) with SYBR Green fluorescence. Thermal cycling was performed with an initial denaturation at 94°C for 3 min, followed by 40 cycles of denaturation at 94°C for 12 s and annealing/extension at 62°C for 30 s. RNA target detection (e.g. SARS-CoV-2) was carried out using an RT-qPCR detection kit under the same cycling conditions.

### Calculation of LOD and DF

The Limit of Detection (LOD) was determined by performing the trans-cleavage assay using varying concentrations of RNA or DNA targets. The LOD value was calculated using the formula 3σ/slope, where σ denotes the standard deviation of three blank measurements. The distinguishing factor (DF) was calculated as: DF = (*F*_WT_ − *F*_WT0_)/(*F*_SNV_ − *F*_SNV0_), where *F*_SNV_ and *F*_WT_ are the fluorescence signals generated by the SNV and WT targets, respectively. *F*_WT0_ and *F*_SNV0_ represent the initial fluorescence signals generated by the SNV and WT targets

### Statistics and reproducibility

No statistical method was used to predetermine sample size. All data were included in the analyses without exclusion. The experiments were not randomized, and the investigators were aware of the group allocations during the experiments and outcome assessments.

## Results

### Construction of PAM-independent and *cis-*cleavage free Cas12a systems

The conventional Cas12a system recognizes both the canonical PAM (TTTV) and suboptimal PAMs such as NTTV and TTTN [38], and shows significantly enhanced performance in one-pot reactions when suboptimal PAMs are employed. However, the use of suboptimal PAMs still imposes sequence constraints and retains partial *cis*-cleavage activity, which can interfere with isothermal amplification processes. Therefore, an ideal Cas12a system for one-pot testing should be independent of PAM requirements and free from *cis*-cleavage activity.

**Figure 1.**
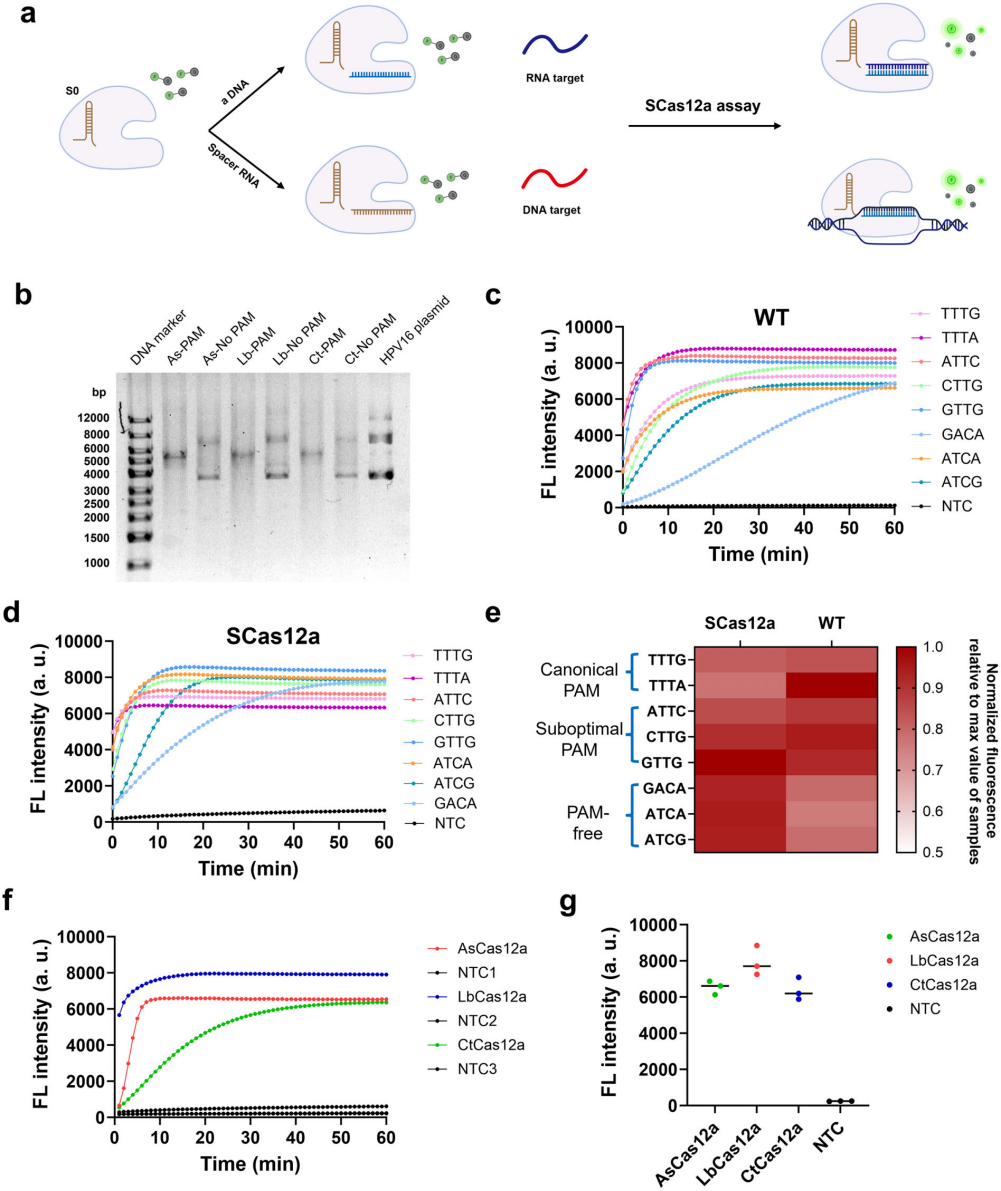
SCas12a system enables PAM- and *cis-*cleavage-free detection of DNA. (**a**) Schematic illustration of the SCas12a assay for detecting RNA and DNA targets. (**b**) Cis-cleavage of target dsDNA plasmids by As, Lb, and Ct Cas12a proteins, with or without a PAM sequence (TTTG). The reactions contained 100 nM Cas12a, 200 nM split crRNA, and 10 nM target dsDNA plasmids containing HPV16 target sequences. (**c**) Fluorescence-based detection of WT Cas12a’s trans-cleavage activity in the presence of target dsDNA substrates containing canonical, suboptimal, or absent PAM sequences, using a complete crRNA targeting the gene fragment of HPV16. The reactions contained 250 nM Cas12a, 500 nM crRNA, 10 nM target dsDNA substrate, and 1000 nM FAM-labeled ssDNA fluorescence probes. (**d**) Fluorescence-based detection of SCas12a system’s trans-cleavage activity in the presence of target dsDNA substrates containing canonical, suboptimal, or absent PAM sequences, using a split crRNA targeting the gene fragment of HPV16. The reactions contained 250 nM Cas12a, 500 nM crRNA, 10 nM target dsDNA substrate, and 1000 nM FAM-labeled ssDNA fluorescence probes. (**e**) Heatmap representing a comparison of the trans-cleavage activity between the SCas12a and WT Cas12a systems on identical DNA substrates with or without PAM sequences. (**f**) Detection of PAM-free dsDNA substrates by As, Lb, and Ct Cas12a proteins. The reaction mixtures were incubated for 60 min at 37°C for AsCas12a and LbCas12a, and at 55°C for CtCas12a, and contained 250 nM Cas12a, 500 nM crRNA, 10 nM target dsDNA substrate, and 1000 nM FAM-labeled ssDNA fluorescence probes. (**g**) Comparison of the maximum fluorescence values among As, Lb, and Ct Cas12a in Fig. 1f. Mean ± s.d. of *n* = 3 technical replicates for panels (c–g). a.u., arbitrary units.

Recently, we developed the SCas12a assay (Fig. 1a), which combines the Cas12a enzyme with a split crRNA composed of scaffold and spacer RNA, enabling direct RNA detection without reverse transcription [36]. In this study, we re-evaluated the SCas12a system’s capability for DNA detection. First, we tested three Cas12a variants, including As, Lb, and Ct Cas12a, and examined their *cis*-cleavage activity on a plasmid (Fig. 1b) with or without a canonical PAM. The “double-band” pattern corresponds to supercoiled and linear forms of the plasmid DNA, confirming that plasmids lacking a PAM remained uncleaved. The “single-band” pattern represents exclusively linear DNA, indicating complete cleavage of plasmids bearing a TTTG PAM. These observations were further validated by qPCR (Supplementary Fig. S1). In this experiment, dsDNA substrates—identical in crRNA-targeting sequence but differing in their PAM sequences—were incubated with AsCas12a RNP complexes formed by WT or SCas12a. Only SCas12a targeting PAM-free substrates yielded Ct values identical to the no-enzyme control, confirming complete loss of *cis*-cleavage activity.

Next, using AsCas12a as an example, we compared the *trans*-cleavage activity of the WT (Fig. 1c) and the SCas12a (Fig. 1d) on dsDNA substrates containing the same 20-bp targeting sequence, but varying in their PAM sequences or the absence thereof. Consistent with previous reports, WT Cas12a showed faster kinetics and stronger fluorescence signals with canonical (TTTG and TTTA) and three suboptimal PAMs [34], whereas PAM-free substrates exhibited the lowest *trans*-cleavage activity and kinetics. In contrast, SCas12a unexpectedly exhibited substantially higher *trans*-cleavage activity against PAM-lacking DNA targets, surpassing even that observed with canonical and suboptimal PAMs in the WT Cas12a system (Fig. 1e). Moreover, this behavior was consistent across all tested Cas12a variants (Fig. 1f–g), indicating that the split-crRNA strategy is universally applicable for PAM-independent and *cis*-cleavage-free DNA detection, laying the foundation for the one-pot CRISPR diagnostics described in the following sections.

### Design principle of the PAM-free DNA detection method

**Figure 2.**
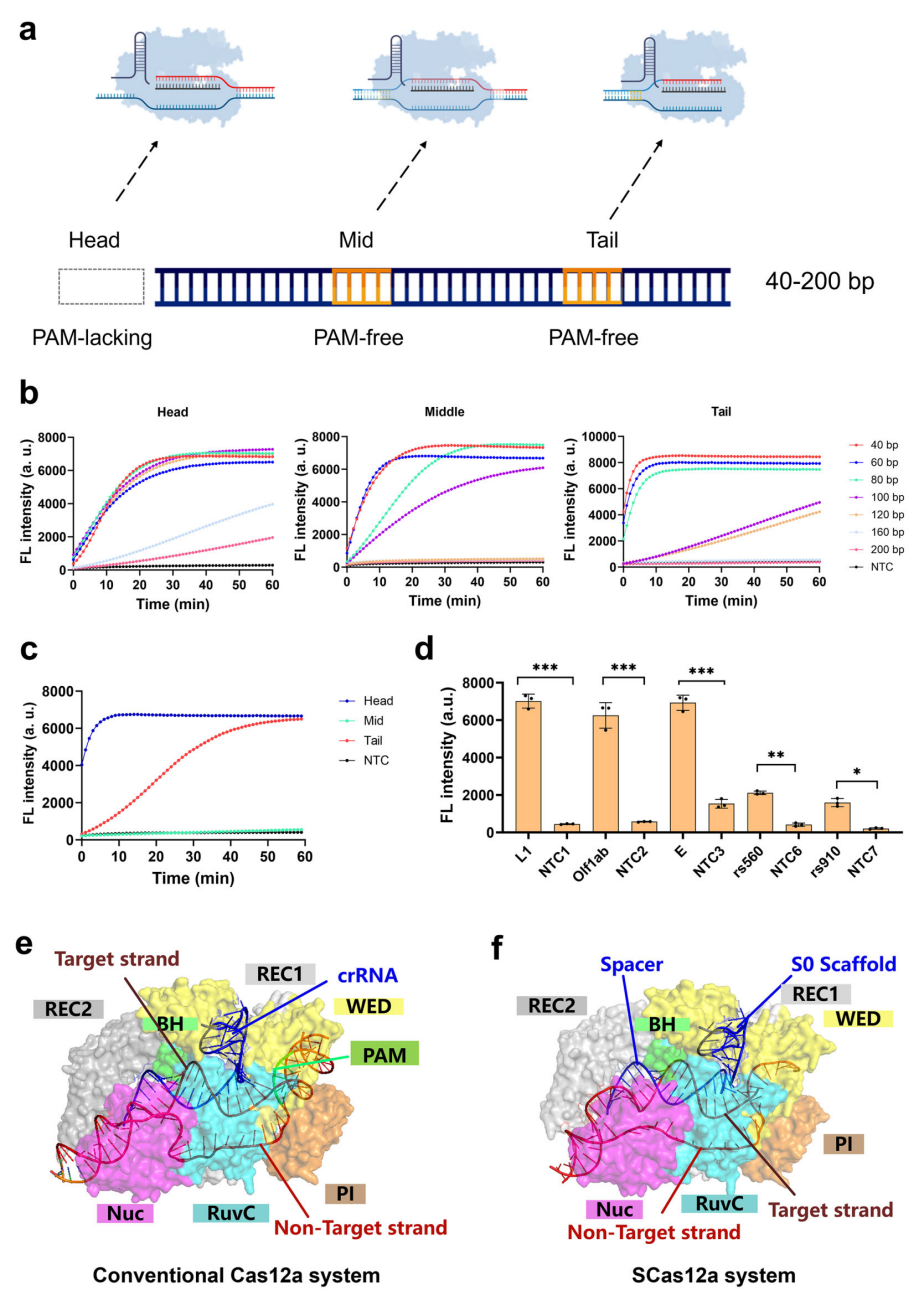
Principle of PAM-free DNA detection using SCas12a. (**a**) Schematic illustration of SCas12a for detecting PAM-free DNA targets at various locations. (**b**) Fluorescence-based detection of SCas12a trans-cleavage activity in the presence of target dsDNA substrates of varying lengths, with PAM-free sites positioned at the head, middle, and tail regions. (**c**) Fluorescence-based detection of SCas12a trans-cleavage activity in the presence of 120-bp HPV16 DNA, with PAM-free sites located at the head, middle, and tail regions. (**d**) Application of the SCas12a assay with head-region design for PAM-free detection of diverse DNA targets. (**e**) Crystal structure of the AsCas12a/crRNA + DNA (with a PAM) complex (PDB:6GQZ). (**f**) Overall structure of the AsCas12a/split crRNA + DNA (without a PAM) predicted by AlphaFold3. Mean ± s.d. of *n* = 3 technical replicates for panels (b–d).

To develop a one-pot CRISPR-based detection assay for trace amounts of nucleic acid molecules, we aimed to establish its design principles and determine the optimal reaction conditions. Initially, we investigated the effects of target length and the location of PAM-free sites on the detection efficiency of the SCas12a system. Accordingly, a series of target DNA fragments ranging from 40 to 200 bp, with PAM-free sites positioned at the head, middle, and tail regions (Fig. 2a), were tested. The results demonstrate that the middle location design showed the slowest kinetics and could not be detected when the length exceeded 120 bp (Fig. 2b). In contrast, the designs with PAM-free sites at the head and tail exhibited faster kinetics and stronger fluorescence signals. Notably, the head location showed the best detection efficiency at 120 bp, which is the optimal targeting length for INA. Furthermore, a parallel comparison of the three models was conducted using an identical 120 bp HPV18 DNA target (Fig. 2c), revealing that the head design demonstrated the fastest reaction kinetics and the highest fluorescence intensity. Accordingly, the design incorporating a PAM-lacking 120 bp DNA target was selected for subsequent development of the one-pot assay, unless otherwise stated.

Next, we evaluated the universality of SCas12a assay by testing it against a variety of DNA targets (Fig. 2d). The results demonstrate that all five selected DNA sequences were efficiently detected. Notably, targets with lower GC content, especially at the 5′-end, showed enhanced detection efficiency. These findings offer valuable insights for guiding the design of subsequent one-pot detection experiments. Finally, using the AlphaFold3 Server, we performed predictive structural modeling to analyze the complex formed by Cas12a with the S0 scaffold and a spacer targeting dsDNA without a PAM (Fig. 2f). The crystal structure of Cas12a in complex with crRNA targeting PAM-containing dsDNA was analyzed (Fig. 2e). The predicted structure of the SCas12a/DNA complex reveals a conformation similar to that observed in the WT Cas12a/DNA complex, enabling efficient access of ssDNA probes to the RuvC domain for *trans*-cleavage. Furthermore, the non-target strand (NTS) without a PAM sequence extends outward, assisting in dsDNA unwinding. However, longer DNA strands exhibit reduced unwinding efficiency (Supplementary Fig. S2), making detection more challenging. Collectively, these findings elucidate the structural basis for PAM-free detection of DNA using the SCas12a system.

### One-pot CRISPR detection by leveraging SCas12a systems

**Figure 3.**
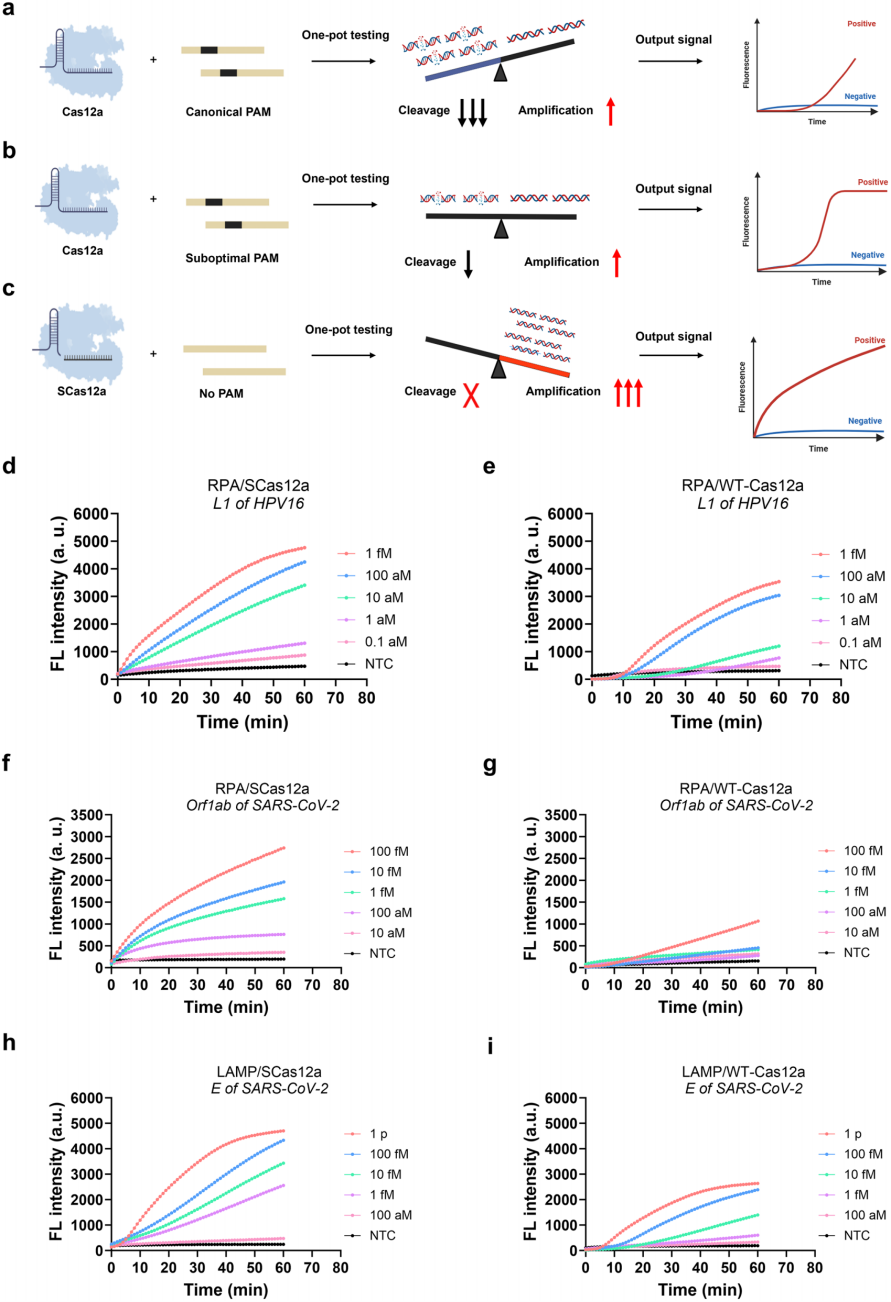
SCas12a design enables faster and more sensitive one-pot testing than WT Cas12a. Schematic depiction of the isothermal amplification and cleavage process in WT-Cas12a and SCas12a-mediated one-pot assays utilizing canonical PAM (**a**), suboptimal PAM (**b**), and no PAM (**c**), respectively. (**d, e**) The L1 gene of HPV16 was selected as the target. The sensitivity of one-pot RPA/CRISPR reactions was compared using a PAM-lacking target with SCas12a (d) and a canonical PAM (TTTA) target with WT-Cas12a (e). (**f, g**) The Orf1ab gene of SARS-Cov-2 was selected as the target. The sensitivity of one-pot RPA/CRISPR reactions was compared using a PAM-lacking target with SCas12a (f) and a canonical PAM (TTTA) target with WT-Cas12a (g). (**h, i**), The E gene of SARS-Cov-2 was selected as the target. The sensitivity of one-pot LAMP/CRISPR reactions was compared using a PAM-lacking target with SCas12a (h) and a canonical PAM (TTTA) target with WT-Cas12a (i). Mean ± s.d. of *n* = 3 technical replicates for panels (d–i).

In the conventional one-pot CRISPR assay utilizing Cas12a and a canonical PAM (Fig. 3a), CRISPR-mediated detection and isothermal amplification of substrates compete with each other. The higher binding affinity conferred by the canonical PAM allows *cis*-cleavage to dominate over amplification, leading to delayed or reduced amplicon generation. In contrast, adjusting the cleavage kinetics by employing a suboptimal PAM for Cas12a (Fig. 3b) can significantly enhance the assay’s detection efficiency due to reduced *cis*-cleavage during amplicon production. In this study, we hypothesized that applying the *cis*-cleavage-free SCas12a system (Fig. 3c) to DNA targets without PAMs would markedly improve the sensitivity, speed, and reproducibility of one-pot detection.

To validate the hypothesis, we first employed the one-pot RPA/SCas12a assay to detect the *L1* gene of HPV16 (Fig. 3d) and the *Orf1ab* gene of SARS-CoV-2 (Fig. 3f), respectively. Meanwhile, WT-Cas12a targeting the identical DNAs but containing the canonical PAM (TTTA) was tested for comparison (Fig. 3e and g). The results showed that, within the first 10 min, the WT-Cas12a exhibited minimal fluorescence signal production. In contrast, the SCas12a exhibited an immediate increase in fluorescence, indicating no interference between RPA and Cas12a during the detection process. Consequently, the RPA/SCas12a assay demonstrated a faster reaction speed and generated significant fluorescence signals over a broader range of target concentrations. This conclusion is further supported by comparative analysis of amplicon profiles generated in the one-pot CRISPR assay using WT Cas12a and SCas12a. As shown in Supplementary Fig. S3, amplification products in the WT Cas12a system exhibit negligible accumulation within 15 min, which is consistent with efficient degradation mediated by its robust *cis*-cleavage activity. In contrast, the SCas12a system shows rapid and pronounced amplicon accumulation beginning at 5 min post-reaction initiation—confirming its lack of *cis-*cleavage activity.

The detection limits of the WT-Cas12a using the canonical PAM were 10 aM (∼6.0 copies/μl) for *L1* and 100 fM (∼6.02 × 10^4^ copies/μl) for *Orf1ab*, respectively. In contrast, the detection limits achieved by the SCas12a were 0.1 aM (0.06 copies/μl) for *L1* and 10 aM (∼6.0 copies/μl) for *Orf1ab*, representing a 100- to 1000-fold improvement in sensitivity, along with a significantly enhanced signal-to-noise ratio (Fig. 3d–g). Furthermore, the reliability of the SCas12a-based assay with PAM-free recognition and the WT-Cas12a-based assay with canonical PAM was evaluated by repeating the experiments under identical conditions ten times, with two replicates per run (Supplementary Fig. S4). The data demonstrate that the fluorescence signals from the SCas12a group were highly consistent across all replicates, whereas those from the WT-Cas12a group exhibited more than a 10-fold variation between replicates.

To broaden the application scope of the SCas12a assay, we further assessed its performance in a LAMP-mediated one-pot detection reaction using the thermostable CtCas12a at its optimal enzymatic temperature of 55°C. The *E* gene of SARS-CoV-2 was selected as the target, and the results demonstrated that the combination of LAMP with SCas12a exhibited significantly accelerated reaction kinetics compared to that with WT-Cas12a (Fig. 3h–i), achieving a 10-fold increase in sensitivity. These findings suggest that SCas12a markedly reduced interference with the LAMP process.

### Detection of HPV16 in clinical samples using RT–RPA/SCas12a

**Figure 4.**
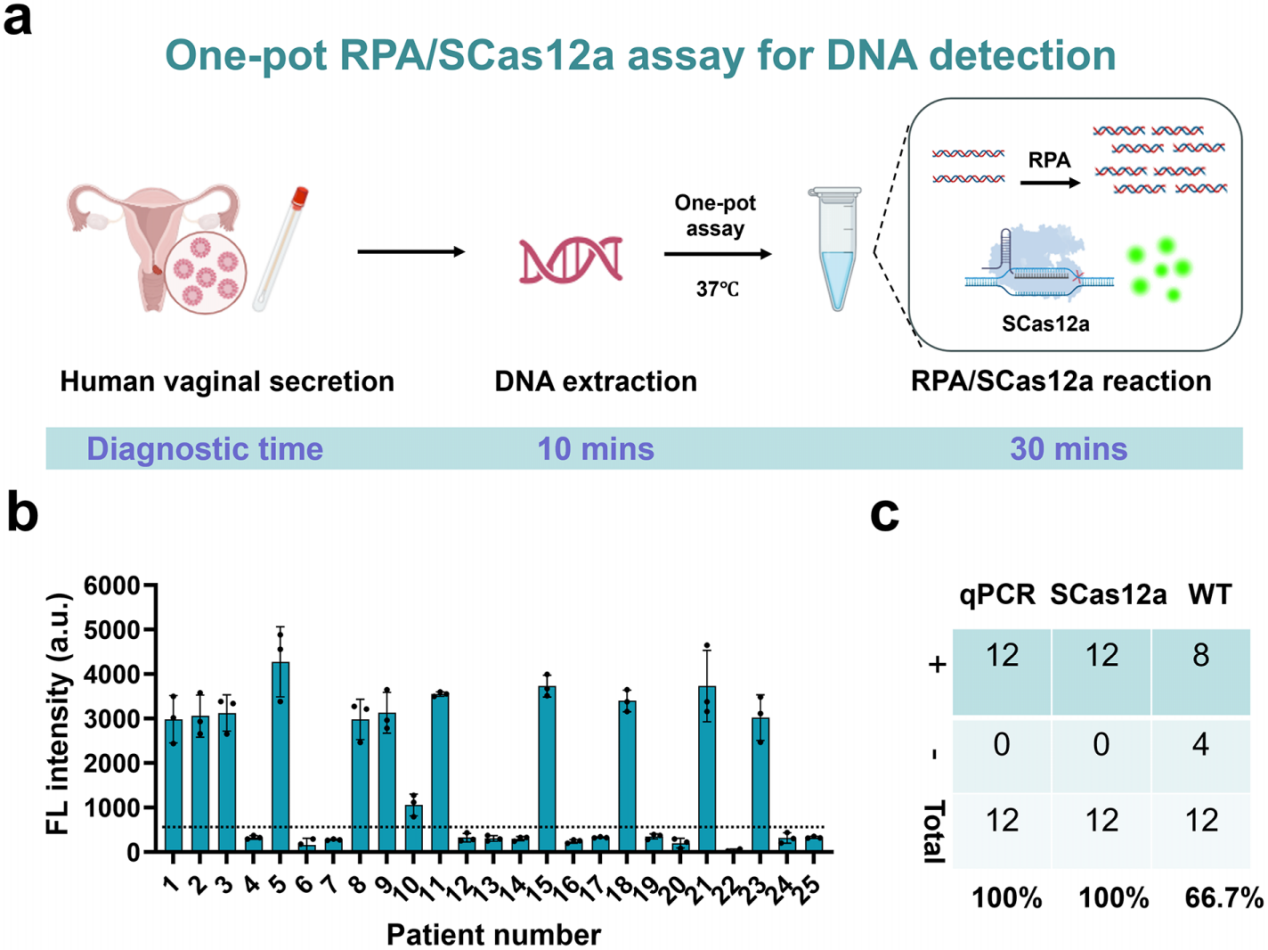
Detetion of HPV16 in clinical samples using RPA/SCas12a assay. (**a**) Schematic diagram of detecting HPV16 from the human vaginal secretion samples by RPA/SCas12a assay. (**b**) Indentification of HPV16 in 25 clinical samples by RPA/SCas12a asasy. Error bars indicate the mean value ± s.d. of three technical replicates. (**c**) Concordance table comparing qPCR and one-pot testing using SCas12a or WT-Cas12a for 12 HPV16-infected samples.

To validate the practical application of the one-pot testing method, we first employed the RPA/SCas12a assay to detect crude DNA extracted from 25 human vaginal secretion samples (Fig. 4a), which had previously been analyzed for HPV16 infection using qPCR (Supplementary Fig. S5). The method accurately identified all 12 HPV16-positive individuals among these clinical samples (Fig. 4b), demonstrating 100% sensitivity and 100% concordance with the qPCR results (Fig. 4c). In contrast, the one-pot CRISPR assay employing the classical DETECTR [10] method with WT Cas12a demonstrated a sensitivity of only 66.7%, detecting 8 out of the 12 samples. In conclusion, these findings demonstrate that SCas12a outperforms DETECTR in one-pot DNA detection assays.

### Detection of SARS-Cov-2 in clinical samples using RT–RPA/SCas12a

**Figure 5.**
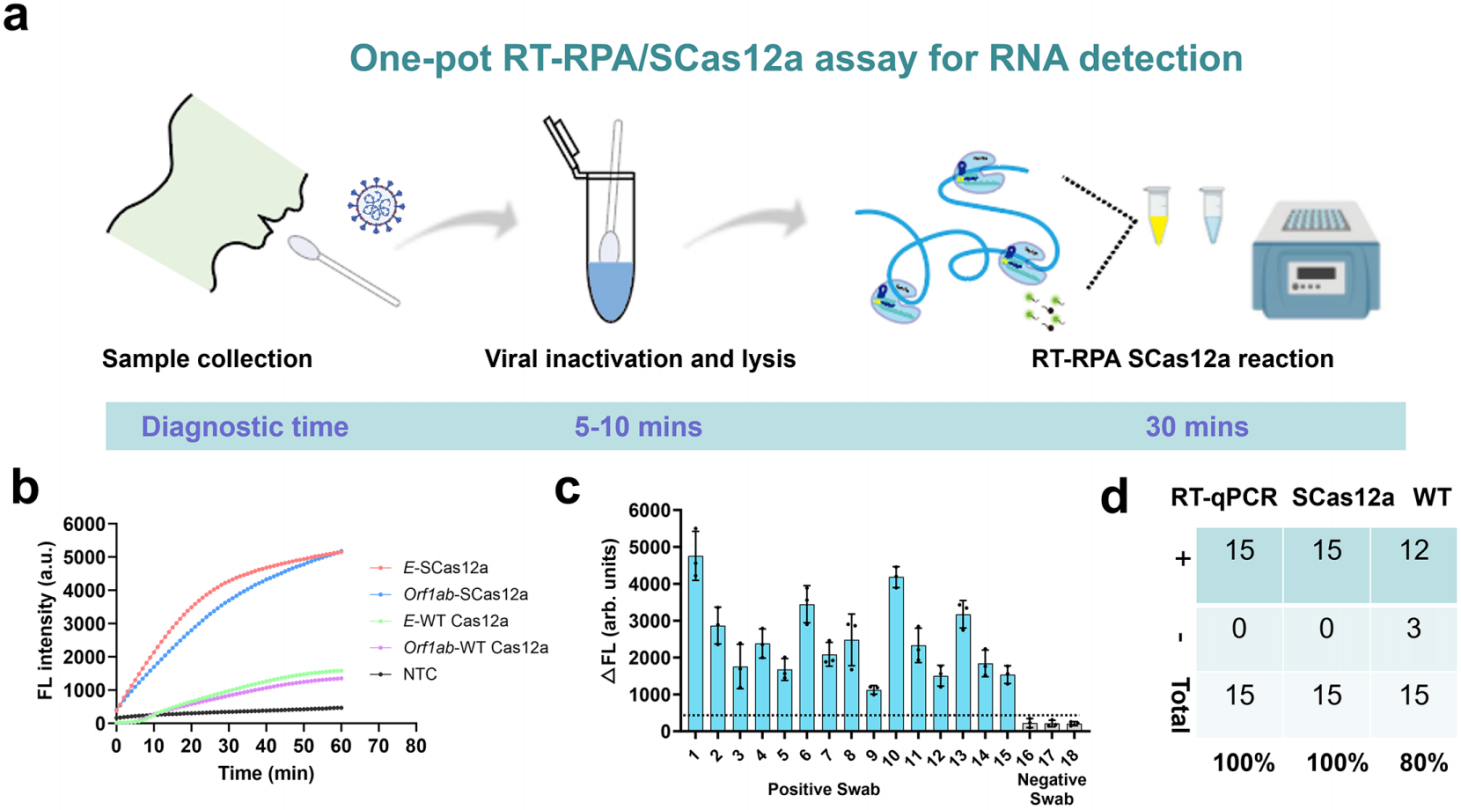
Detection of SARS-Cov-2 in clinical samples using RT–RPA/SCas12a assay. (**a**) Schematic diagram of detecting SARS-Cov-2 from the clinical swab samples by RT–RPA/SCas12a assay. (**b**) Fluorescence signals of one-pot testing using spacers targeting the Orf1ab and E genes of SARS-Cov-2. (**c**) The RNA from 15 nasopharyngeal swabs confirmed positive for SARS-CoV-2 by RT-qPCR was tested by RT–RPA/SCas12a assay. Three confirmed negative swabs were tested for comparison. Error bars indicate the mean value ± s.d of three technical replicates. (**d**) Concordance table comparing RT-qPCR and one-pot testing using SCas12a or WT Cas12a for 15 SARS-Cov-2-infected samples.

To further verify the applicability of one-pot testing for pathogen-induced infections, we assessed the performance of RT–RPA/SCas12a using clinical specimens from individuals infected with SARS-CoV-2. The complete testing workflow is illustrated in Fig. 5a and requires ~40 min, comprising 5–10 min for viral inactivation and lysis by heating, followed by a 30-min RT–RPA/SCas12a reaction. The *E* and *Orf1ab* genes of SARS-CoV-2 were specifically detected (Fig. 5b) by SCas12a and WT Cas12a, with the former demonstrating at least a 10-fold higher detection efficiency. Subsequently, we applied the RT–RPA/SCas12a assay (Fig. 5c) to fifteen nasal swab specimens collected from individuals confirmed to have SARS-CoV-2 infection via clinical RT-qPCR analysis. These samples exhibited Ct values ranging from 11.59 to 27.23 (Supplementary Fig. S6). All fifteen samples were correctly identified as positive, showing a clear distinction in amplification slopes compared to three negative control swabs, resulting in a sensitivity of 100% (Fig. 5d). In contrast, the RT–RPA/WT-Cas12a achieved a sensitivity of 80%, detecting only 12 out of the 15 samples. Collectively, these findings demonstrate that SCas12a outperforms WT-Cas12a in one-pot diagnostics for RNA detection.

### Single-nucleotide specificity of RPA/SCas12a for human genotyping

**Figure 6.**
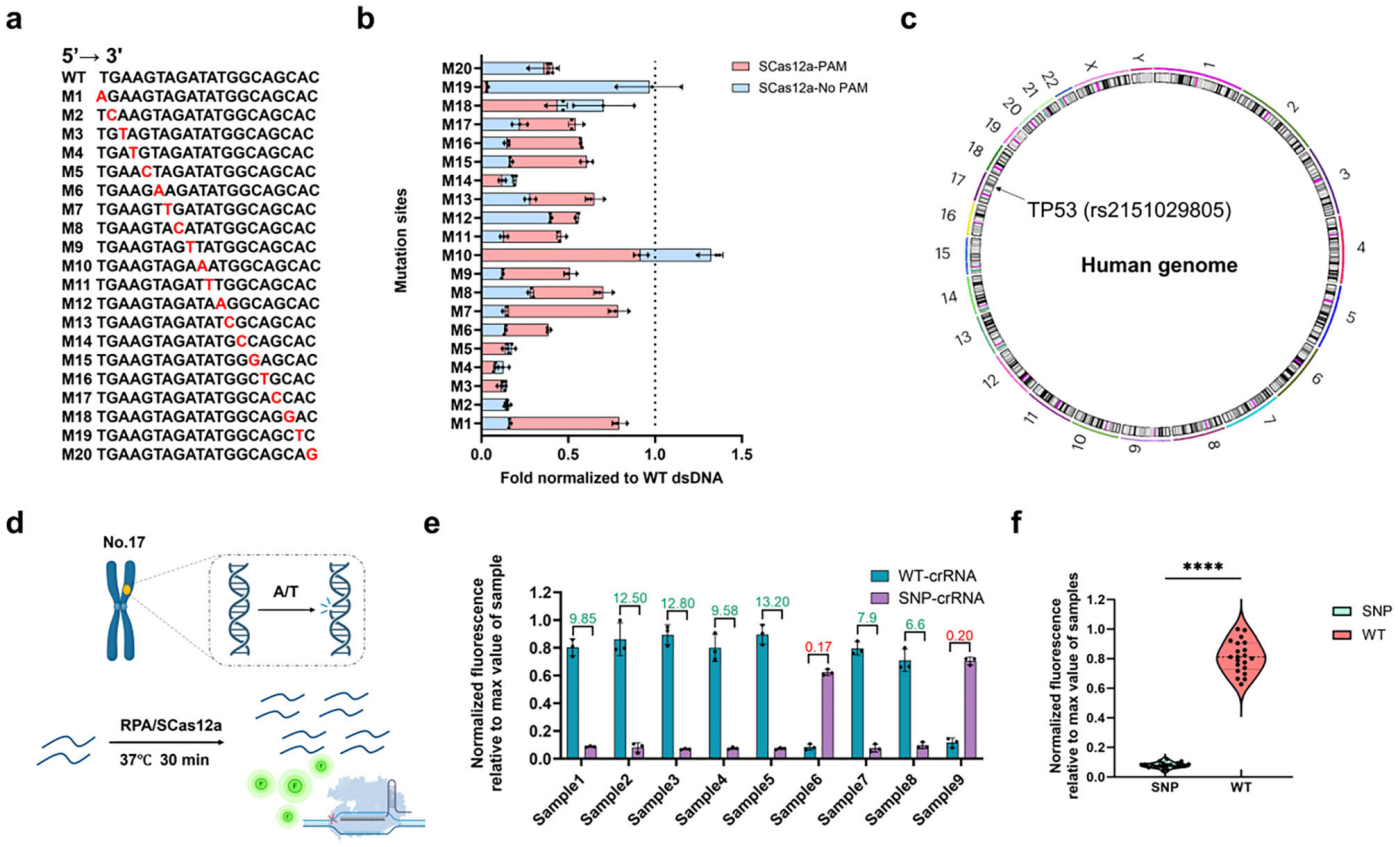
RPA/SCas12a assay can discriminate SNPs in human genotyping. (**a**) Specificity of SCas12a toward single point mutations in a dsDNA target without a PAM. Spacer RNA activators were designed with point mutations spanning the entire pairing region of an HPV16-derived DNA target. The mutation position is indicated by “M,” where the base has been substituted with its complementary deoxynucleotide. (**b**) Comparison of fluorescence changes for the trans-cleavage activity of SCas12a between DNA substrates with and without a PAM. (**c**) Circos plot illustrating the genomic locations of human TP53 (rs2151029805) SNPs detected using the one-pot testing method. (**d**) Schematic illustration of RPA/SCas12a assay for human genotyping. (**e**) Detection of nine different oral squamous cell corcinoma (OSCC) individuals at the TP53 SNP site (A > T mutation) in the human genome using the RPA/SCas12a assay with spacer RNAs containing a single mismatch. The DF values were calculated. (**f**) Identification of TP53 SNPs in nine clinical samples by one-pot testing. Data are represented as the mean value ± standard deviation of three technical replicates.

We and other groups have demonstrated that the SCas12a system exhibits significantly enhanced specificity for mutations located in the PAM-distal regions of DNA targets [36, 39, 40]. Compared to DNA with PAMs, the split crRNA is expected to show reduced affinity toward PAM-free DNA substrates, thereby amplifying the energetic penalty for single-nucleotide mismatches through nonequilibrium hybridization-driven regulation. Accordingly, we harnessed the SCas12a to test fully complementary dsDNA targets with single-nucleotide mismatches at positions 1–20 at a concentration of 1 nM (Fig. 6a). Compared to DNA subsrtate with a PAM, an obviously enhanced specificity was observed across positions 1 to 17, except for positions 10 and 19 (Fig. 6b). The highest DF for this assay was 91-fold, which indicates excellent single-nucleotide specificity in dsDNA detection.

The high single-nucleotide specificity makes SCas12a a powerful tool for rapid human genotyping. In this study, we validated the practical utility of the RPA/SCas12a assay in detecting the cancer-associated TP53 (rs215029805) SNP (A > T) [41] (Fig. 6c). We benchmarked the one-pot detection assay against genotyping results obtained from Sanger sequencing, which is the established gold standard for SNP indentification. Specifically, we obtained nine tissue samples from OSCC patients (seven WT and two A > T mutant samples) and applied the RPA/SCas12a system for SNP detection. The results showed a statistically significant difference in signal intensity between the WT and mutant samples (Fig. 6e). To further validate the reliability of the method, we introduced mutant-specific spacers for reverse confirmation of the mutant samples. These results demonstrated 100% concordance with Sanger sequencing data (Supplementary Fig. S7). The entire one-pot detection process could be completed within 30 min, which is substantially faster than SHERLOCK^11^ (>2 h). Moreover, we established a standard for discriminating WT (DF > 4) and mutant type (DF < 0.25) using DF values.

## Discussion

The current global demand for rapid, ultrasensitive, and instrument-free nucleic acid diagnostics has revealed a fundamental challenge: the very nuclease activity that grants CRISPR systems their remarkable specificity can interfere with the isothermal amplification that provides their target substrate. This “*cis*-cleavage paradox” has necessitated trade-offs between speed, sensitivity, and simplicity in one-pot assays. Although recent elegant one-pot CRISPR studies have mitigated *cis*-cleavage through suboptimal PAMs [34], small-molecule inhibitors such as heparin [35], or light-gated crRNAs [29–33], these strategies still allow residual collateral damage, as the nuclease remains inherently capable of cleaving DNA. Here, we resolve this paradox by re-engineering the surveillance complex rather than merely suppressing its activity. The SCas12a architecture, which features a split crRNA that renders Cas12a PAM-independent and *cis*-cleavage-free, transforms the reaction into a single-turnover burst, effectively decoupling signal generation from target degradation. The result is a 100- to 1000-fold improvement in sensitivity and a >10-fold reduction in time-to-signal compared to canonical Cas12a (Fig. 3d–i), while eliminating the stochastic variability that has hindered previous one-pot CRISPR platforms. A comprehensive comparison with existing one-pot CRISPR-based detection systems is provided in Supplementary Table S2.

Structural analysis indicates that the Cas12a enzyme engages in *trans*-cleavage of the reporter only after stable R-loop formation in the SCas12a system (Fig. 2f). We also demonstrate that the split crRNA lowers the activation energy for *trans*-cleavage while increasing it for *cis*-cleavage, effectively creating a molecular diode that enables signal amplification without target degradation in one-pot reactions. Growing evidence indicates that Cas12a’s *trans*-cleavage activity can be uncoupled from its *cis*-cleavage activity. Specifically, binding of the Cas12a RNP to the target strand of dsDNA is sufficient to trigger robust *trans*-cleavage—even without *cis*-cleavage—provided the target strand is accessible. Binding affinity and *trans*-cleavage magnitude correlate positively. When the dsDNA target contains a canonical PAM, Cas12a RNP exhibits intrinsic helicase-like activity, enabling efficient unwinding and stable R-loop formation; this permits full spacer RNA hybridization with the complementary ssDNA strand and yields strong, length-independent *trans*-cleavage. In contrast, in the absence of a canonical PAM, as in SCas12a-mediated targeting, the RNP lacks helicase activity. Spacer RNA binding then proceeds via strand invasion into intact dsDNA, a process highly sensitive to duplex stability and geometry. Consequently, long dsDNA substrates (>120 bp) resist strand displacement due to structural rigidity, resulting in markedly attenuated *trans*-cleavage; only shorter dsDNA (<120 bp) permits efficient invasion and thus supports measurable, length-dependent *trans*-cleavage activity.

Furthermore, we identify two key determinants of target detection efficiency in the SCas12a system: (i) spacer RNA secondary structure and (ii) target DNA GC content. First, NUPACK-based structural simulations of spacer sequences reveal heterogeneous folding landscapes—increasing structural complexity (e.g. stable hairpins or pseudoknots) impedes Cas12a RNP binding, thereby reducing activation kinetics and downstream trans-cleavage–driven fluorescence signal. Second, higher GC content in the target dsDNA correlates strongly with diminished detection sensitivity. This is mechanistically attributable to the absence of intrinsic helicase activity in SCas12a: target recognition relies on direct strand invasion by the spacer RNA into intact dsDNA, a process thermodynamically disfavored by high-GC duplex stability. Consequently, to maximize assay performance, spacer design should prioritize low structural propensity, and target selection should avoid GC-rich regions (>60%).

The universality of SCas12a desgin is supported by its successful application across three Cas12a orthologs (As, Lb, and Ct) and multiple isothermal amplification approaches (RPA, LAMP, and RT–RPA). Additionally, the RPA/SCas12a assay achieves single-base resolution at nearly all tested detection sites (Fig. 6a and b). At last, we successfully applied the one-pot CRISPR diagnostics to detect HPV16 (Fig. 4b and c), SARS-CoV-2 (Fig. 5b–d), and TP53 SNPs (Fig. 6e and f) in clinical samples.

In summary, we develop a PAM-independent and *cis*-cleavage-free SCas12a system for rapid one-pot nucleic acid detection. This system offers superior speed, sensitivity, and specificity compared to existing one-pot CRISPR diagnostics. Looking forward, the split-crRNA strategy utilized in this study is not limited to the Cas12a family. Preliminary data suggest that an analogous architecture can be grafted onto thermostable Cas12b [42] and LbuCas13a [43], paving the way for a unified, orthogonal CRISPR toolbox in which each effector interrogates a distinct nucleic acid class within a single, closed tube. We therefore anticipate that SCas12a will function as a universal molecular scaffold for decentralized diagnostics, accelerating infectious disease surveillance, enabling precision oncology at the bedside, and democratizing environmental pathogen monitoring with the operational simplicity required for true point-of-care deployment.

## Supplementary Material

gkag267_Supplemental_File

## Data Availability

The data underlying this article are available in the article and its online supplementary material.

## References

[1] Larremore DB, Wilder B, Lester E et al. Test sensitivity is secondary to frequency and turnaround time for COVID-19 screening. Sci Adv. 2021;7:eabd5393. 10.1126/sciadv.abd5393. 33219112 PMC7775777

[2] McDermott A . Inner workings: researchers race to develop in-home testing for COVID-19, a potential game changer. Proc Natl Acad Sci USA. 2020;117:25956–9. 10.1073/pnas.2019062117. 32999063 PMC7584891

[3] Hu B, Guo H, Zhou P et al. Characteristics of SARS-CoV-2 and COVID-19. Nat Rev Micro. 2021;19:141–54. 10.1038/s41579-020-00459-7. PMC7537588 33024307

[4] Zhu N, Zhang D, Wang W et al. A novel coronavirus from patients with pneumonia in China, 2019. N Engl J Med. 2020;382:727–33. 10.1056/NEJMoa2001017. 31978945 PMC7092803

[5] Chen Y, Wang R, Gilby N et al. Emerging SARS-CoV-2 variants: why, how, and what’s next?. Cell Insight. 2022;1:100029. 10.1016/j.cellin.2022.100029. 37193049 PMC9057926

[6] Piepenburg O, Williams CH, Stemple DL et al. DNA detection using recombination proteins. PLoS Biol. 2006;4:e204. 10.1371/journal.pbio.0040204. 16756388 PMC1475771

[7] Notomi T, Okayama H, Masubuchi H et al. Loop-mediated isothermal amplification of DNA. Nucleic Acids Res. 2000;28:63e. 10.1093/nar/28.12.e63. PMC102748 10871386

[8] Rolando JC, Jue E, Barlow JT et al. Real-time kinetics and high-resolution melt curves in single-molecule digital LAMP to differentiate and study specific and non-specific amplification. Nucleic Acids Res. 2020;48:e42. 10.1093/nar/gkaa099. 32103255 PMC7144905

[9] Joung J, Ladha A, Saito M et al. Detection of SARS-CoV-2 with SHERLOCK one-pot testing. N Engl J Med. 2020;383:1492–4. 10.1056/NEJMc2026172. 32937062 PMC7510942

[10] Chen JS, Ma E, Harrington LB et al. CRISPR-Cas12a target binding unleashes indiscriminate single-stranded DNase activity. Science. 2018;360:436–9. 10.1126/science.aar6245. 29449511 PMC6628903

[11] Gootenberg JS, Abudayyeh OO, Lee JW et al. Nucleic acid detection with CRISPR-Cas13a/C2c2. Science. 2017;356:438–42. 10.1126/science.aam9321. 28408723 PMC5526198

[12] Bravo JPK, Li T, Liu H et al. RNA targeting unleashes indiscriminate nuclease activity of CRISPR-Cas12a2. Nature. 2023;613:582–7. 10.1038/s41586-022-05560-w. 36599980 PMC9849127

[13] Chen J, Chen Y, Huang L et al. Trans-nuclease activity of Cas9 activated by DNA or RNA target binding. Nat Biotechnol. 2025;43:558–68. 10.1038/s41587-024-02255-7. 38811761

[14] Li SY, Cheng QX, Wang JM et al. CRISPR-Cas12a-assisted nucleic acid detection. Cell Discov. 2018;4:20. 10.1038/s41421-018-0028-z. 29707234 PMC5913299

[15] Malcı K, Walls LE, Rios-Solis L. Rational design of CRISPR/Cas12a-RPA based one-pot COVID-19 detection with design of experiments. ACS Synth Biol. 2022;11:1555–67. 10.1021/acssynbio.1c00617. 35363475 PMC9016756

[16] Feng W, Peng H, Xu J et al. Integrating reverse transcription recombinase polymerase amplification with CRISPR technology for the one-tube assay of RNA. Anal Chem. 2021;93:12808–16. 10.1021/acs.analchem.1c03456. 34506127

[17] Xu Z, Chen D, Li T et al. Microfluidic space coding for multiplexed nucleic acid detection via CRISPR-Cas12a and recombinase polymerase amplification. Nat Commun. 2022;13:6480. 10.1038/s41467-022-34086-y. 36309521 PMC9617605

[18] Ooi KH, Liu MM, Tay JWD et al. An engineered CRISPR-Cas12a variant and DNA–RNA hybrid guides enable robust and rapid COVID-19 testing. Nat Commun. 2021;12:1739. 10.1038/s41467-021-21996-6. 33741959 PMC7979722

[19] Li L, Li S, Wu N et al. HOLMESv2: a CRISPR-Cas12b-assisted platform for nucleic acid detection and DNA methylation quantitation. ACS Synth Biol. 2019;8:2228–37. 10.1021/acssynbio.9b00209. 31532637

[20] Joung J, Ladha A, Saito M et al. Detection of SARS-CoV-2 with SHERLOCK one-pot testing. N Engl J Med. 2020;383:1492–4. 10.1056/NEJMc2026172. 32937062 PMC7510942

[21] Li T, Hu R, Xia J et al. G-triplex: a new type of CRISPR-Cas12a reporter enabling highly sensitive nucleic acid detection. Biosens Bioelectron. 2021;187:113292. 10.1016/j.bios.2021.113292. 33991961

[22] Mahas A, Marsic T, Lopez-Portillo Masson M et al. Characterization of a thermostable Cas13 enzyme for one-pot detection of SARS-CoV-2. Proc Natl Acad Sci USA. 2022;119:e2118260119. 10.1073/pnas.2118260119. 35763567 PMC9282225

[23] Nguyen LT, Macaluso NC, Pizzano BL et al. A thermostable Cas12b from *Brevibacillus* leverages one-pot discrimination of SARS-CoV-2 variants of concern. EBioMedicine. 2022;77:103926. 10.1016/j.ebiom.2022.103926. 35290826 PMC8917962

[24] Ding X, Yin K, Li Z et al. Ultrasensitive and visual detection of SARS-CoV-2 using all-in-one dual CRISPR-Cas12a assay. Nat Commun. 2020;11:4711. 10.1038/s41467-020-18575-6. 32948757 PMC7501862

[25] Yang L, Chen G, Wu J et al. A PAM-free one-step asymmetric RPA and CRISPR/Cas12b combined assay (OAR-CRISPR) for rapid and ultrasensitive DNA detection. Anal Chem. 2024;96:5471–7. 10.1021/acs.analchem.3c05545. 38551977

[26] Zhao L, Wang H, Chen X et al. Agarose hydrogel-boosted one-tube RPA-CRISPR/Cas12a assay for robust point-of-care detection of zoonotic nematode *Anisakis*. J Agric Food Chem. 2024;72:8257–68. 10.1021/acs.jafc.4c00204. 38530904

[27] Lin M, Yue H, Tian T et al. Glycerol additive boosts 100-fold sensitivity enhancement for one-pot RPA-CRISPR/Cas12a assay. Anal Chem. 2022;94:8277–84. 10.1021/acs.analchem.2c00616. 35635176

[28] Yin K, Ding X, Li Z et al. Dynamic aqueous multiphase reaction system for one-pot CRISPR-Cas12a-based ultrasensitive and quantitative molecular diagnosis. Anal Chem. 2020;92:8561–8. 10.1021/acs.analchem.0c01459. 32390420 PMC7588651

[29] Hu M, Liu R, Qiu Z et al. Light-start CRISPR-Cas12a reaction with caged crRNA enables rapid and sensitive nucleic acid detection. Angew Chem Int Ed. 2023;62:e202300663. 10.1002/anie.202300663. 37016515

[30] Hu M, Qiu Z, Bi Z et al. Photocontrolled crRNA activation enables robust CRISPR-Cas12a diagnostics. Proc Natl Acad Sci USA. 2022;119:e2202034119. 10.1073/pnas.2202034119. PMC9245704 35727982

[31] Hu M, Liu R, Qiu Z et al. Photocontrolled programmable enzymatic cascade for robust CRISPR diagnostics. J Am Chem Soc. 2025;147:31004–15. 10.1021/jacs.5c01234. 40802893

[32] Tian T, Xiao H, Guo X et al. Identification of a key nucleotide influencing Cas12a crRNA activity for universal photo-controlled CRISPR diagnostics. Nat Commun. 2025;16:6694. 10.1038/s41467-025-62082-5. 40691444 PMC12279986

[33] Hu M, Liu R, Qiu Z et al. Scalable modulation of CRISPR-Cas enzyme activity using photocleavable phosphorothioate DNA. Nat Commun. 2025;16:5939. 10.1038/s41467-025-62081-6. 40593724 PMC12216424

[34] Lu S, Tong X, Han Y et al. Fast and sensitive detection of SARS-CoV-2 RNA using suboptimal protospacer adjacent motifs for Cas12a. Nat Biomed Eng. 2022;6:286–97. 10.1038/s41551-022-00861-x. 35314803

[35] Cheng Z, Luo X, Yu S et al. Tunable control of Cas12 activity promotes universal and fast one-pot nucleic acid detection. Nat Commun. 2025;16:1166. 10.1038/s41467-025-56516-3. 39885211 PMC11782535

[36] Chen Y, Wang X, Zhang J et al. Split crRNA with CRISPR-Cas12a enabling highly sensitive and multiplexed detection of RNA and DNA. Nat Commun. 2024;15:8342. 10.1038/s41467-024-52691-x. 39333528 PMC11436650

[37] Zhang J, Yin W, Jiang Q et al. Precise amplification-free detection of highly structured RNA with an enhanced SCas12a assay. Commun Biol. 2025;8:366. 10.1038/s42003-025-07104-0. 40038432 PMC11880562

[38] Yamano T, Zetsche B, Ishitani R et al. Structural basis for the canonical and non-canonical PAM recognition by CRISPR-Cpf1. Mol Cell. 2017;67:633–45. 10.1016/j.molcel.2017.06.035. 28781234 PMC5957536

[39] Rananaware SR, Vesco EK, Shoemaker GM et al. Programmable RNA detection with CRISPR-Cas12a. Nat Commun. 2023;14:5409. 10.1038/s41467-023-41006-1. 37669948 PMC10480431

[40] Liu Q, Jiang Z, Li S et al. Nonequilibrium hybridization-driven CRISPR/Cas adapter with extended energetic penalty for discrimination of single-nucleotide variants. Nucleic Acids Res. 2025;53:gkaf287. 10.1093/nar/gkaf287. 40243059 PMC12004117

[41] Cho Y, Gorina S, Jeffrey PD et al. Crystal structure of a p53 tumor suppressor–DNA complex: understanding tumorigenic mutations. Science. 1994;265:346–55. 10.1126/science.8023157. 8023157

[42] Tong X, Zhang K, Han Y et al. Fast and sensitive CRISPR detection by minimized interference of target amplification. Nat Chem Biol. 2024;20:885–93. 10.1038/s41589-023-01534-9. 38332130

[43] Wu X, Luo S, Guo C et al. LbuCas13a directly targets DNA and elicits strong trans-cleavage activity. Nat Biomed Eng. 2025;9:2141–54. 10.1038/s41551-025-01424-6. 40542106

