# Supplementary material for: Elimination of cis-cleavage in CRISPR diagnostics for one-pot rapid nucleic acid detection
Source: Nucleic Acids Res. 2026 Mar 26;54(6):gkag267. doi: 10.1093/nar/gkag267 (PMC13019291; doi:10.1093/nar/gkag267)
Supplement: gkag267_Supplemental_File [file gkag267_supplemental_file.pdf]

Supporting information

Elimination of *cis*-cleavage in CRISPR diagnostics for one-pot rapid nucleic acid detection

Wenhao Yin[1, 2+], Zhili Jin[3+], Qingyuan Jiang[1+], Shuqi Jin[1], Xinping Wang[1], Ruyi He[2], Bin Qiao[4], Jie Qiao[2*], Xianhua Zhang[1*] and Yi Liu[1, 5*]

[1] State Key Laboratory of Biocatalysis and Enzyme Engineering, School of Life Sciences, Hubei University, Hubei 430042 (China)

[2] School of Life Science and Technology, Wuhan Polytechnic University, Hubei 430023 (China)

[3] Department of Cardiology, Zhongnan Hospital of Wuhan University, Hubei 430071 (China)

[4] Department of Oral and Maxillofacial Surgery, The First Affiliated Hospital of Zhengzhou University, Zhengzhou University, Zhengzhou 450001 (China)

[5] BravoVax Co., Ltd., Wuhan, Hubei 430075 (China)

+ There authors contributed equally to this work.

*To whom correspondence should be addressed to Jie Qiao (jieqiao@whpu.edu.cn); Xianhua Zhang (xhzhang0072@hubu.edu.cn); Yi Liu (yiliu0825@hubu.edu.cn).

Table S1. The sequences of the nucleic acids used in this study.

**RPA and RT-RPA primers**	**Sequence**
HPV16-RPA-F	TGGGGTAACCAACTATTTGTTACTGTT
HPV16-RPA-R	TCATATTCGCTCCCATGTCG
Orf1ab-RPA-F	CTAAAGCTTACAAAGATTATCTAGCTAGTGG
Orf1ab-RPA-R	CAGTACAGACAACACGATGCACCA
TP53-RPA-F	TGCTCAGATAGCGATGGTGAGCA
TP53-RPA-R	ATGCTGAGGAGGGGCCAGACCTAAGA
E gene-RPA-F	ATGTACTCATTCGTTTCGGAAGAGACAGGTA
E gene-RPA-F	GAAGGTTTTACAAGACTCACGTTAACAA
RT-Orf1ab	TTAGCACAAGTTGTAGGT
RE-E gene	TAATATAATATTTAGTTCGT
**LAMP primers**	**Sequence**
E-F3	TGAGTACGAACTTATGTACTCAT
E-B3	TTCAGATTTTTAACACGAGAGT
E-FIP	ACCACGAAAGCAAGAAAAAGAAGTTCGTTTCGGA AGAGACAG
E-BIP	TTGCTAGTTACACTAGCCATCCTTAGGTTTTACAAG ACTCACGT
E-Loop-F	CGCTATTAACTATTAACG
E-Loop-B	GCGCTTCGATTGTGTGCGT
**crRNA sequences**	**Sequence**
Scaffold	AAUUUCUACUAAGUGUAGAU
HPV16-L1-spacer	UGGGGUAACCAACUAUUUGU
Orf1ab-spacer	CUAAAGCUUACAAAGAUUAU
E-spacer	AUGUACUCAUUCGUUUCGGA
S-spacer	AGGUUUCAAACUUUACUUGC
E6-spacer	AUUAGAAUGUGUGUACUGCA
rs560-spacer	UCUUCUGUUCUCAAAGCAUC
rs910-spacer	CGAGGUCCAGAGAUACAUUG
TP53-spacer	UGCUCAGAUAGCGAUGGUGA
TP53-SNP-spacer	UGCUCAGAUAGCGUUGGUGA
HPV16-crRNA	AAUUUCUACUAAGUGUAGAUUGAAGUAGAUAUG GCAGCAC
Orf1ab-crRNA	AAUUUCUACUAAGUGUAGAUCAACCAAUCACUAA UUGUGU
**PAM and target sequences**	**non-target and target strands with PAMs**
HPV16-target-1	NTS: 5’- TTTATGAAGTAGATATGGCAGCAC -3’<br>TS: 3’- AAATCACGACGGTATAGATGAAGT-5’
HPV16-target-2	NTS: 5’- TTTGTGAAGTAGATATGGCAGCAC -3’<br>TS: 3’- AAACCACGACGGTATAGATGAAGT-5’
HPV16-target-3	NTS: 5’- ATTCTGAAGTAGATATGGCAGCAC -3’

	TS: 3'- TAAGCACGACGGTATAGATGAAGT-5'
HPV16-target-4	NTS: 5'- CTTGTGAAGTAGATATGGCAGCAC -3'<br>TS: 3'- GAACCACGACGGTATAGATGAAGT-5'
HPV16-target-5	NTS: 5'- GTTGTGAAGTAGATATGGCAGCAC -3'<br>TS: 3'- CAACCACGACGGTATAGATGAAGT-5'
HPV16-target-6	NTS: 5'- ATCATGAAGTAGATATGGCAGCAC -3'<br>TS: 3'- TAGTCACGACGGTATAGATGAAGT-5'
HPV16-target-7	NTS: 5'- ATCGTGAAGTAGATATGGCAGCAC -3'<br>TS: 3'- TAGCCACGACGGTATAGATGAAGT-5'
HPV16-target-8	NTS: 5'- GACATGAAGTAGATATGGCAGCAC -3'<br>TS: 3'- CTGCCACGACGGTATAGATGAAGT-5'
Target-head	NTS: 5'- CTAAAGCTTACAAAGATTAT -3'<br>TS: 3'- GATTTCGAATGTTTCTAATA-5'
Target-mid	NTS: 5'- ATCACTAAAGCTTACAAAGATTAT -3'<br>TS: 3'- TAGCGATTTCGAATGTTTCTAATA-5'
Target-tail	NTS: 5'- ATCACTAAAGCTTACAAAGATTAT -3'<br>TS: 3'- TAGCGATTTCGAATGTTTCTAATA-5'

Table S2. Comparison of the one-pot CRISPR/Cas12a assays.

Method name	Cas12 variants	Amplification methods	Time (min)	LoD (copies/μL)	PAM requirements
SCas12a (This work）	AsCas12a, LbCas12a, CtCas12a	RPA-37°C	10-20		PAM-free
sPAMC[1]	LbCas12a	RPA-37°C	15-20	0.6	Suboptimal PAMS
SURVEY[2]	LbCas12a, AsCas12a	RPA-37°C	15-20	0.6	Classic and suboptimal PAMS
EXTRA-CRISPR[3]	LbCas12a	RPA-37°C	20-180	About 3000	Classic PAMs
One-pot (DOE)[4]	LbCas12a	RPA-37°C	90	0.5-2	Classic PAMs
One-pot (Photo-controlled)[5]	LbCas12a	RPA-37°C	90	1-10	Classic PAMs
One-pot (Photo-activatable)[6]	LbCas12a	RPA-37°C	40	10	Classic PAMs
One-pot (Glycerol)[7]	LbCas12a	RPA-37℃	60	0.6	Classic PAMs
One-pot (Separated layer)[8]	LbCas12a	RPA-37°C	93	2.5	Classic PAMs
LAMP-Cas12b[9]	AapCas12b	LAMP-61°C	15	0.5	Classic PAMs
CoLAMP[10]	AapCas12b	LAMP-60°C	40	0.5	Classic PAMs

References:

[1] Lu S, Tong X, Han Y et al. Fast and sensitive detection of SARS-CoV-2 RNA using suboptimal protospacer adjacent motifs for Cas12a. *Nat Biomed Eng* 2022;**6:**286–97. https://doi.org/10.1038/s41551-022-00861-x

[2] Cheng ZH, Luo XY, Yu SS et al. Tunable control of Cas12 activity promotes universal and fast one-pot nucleic acid detection. *Nat Commun* 2025;**16:**1166. https://doi.org/10.1038/s41467-025-56516-3

[3] Yan H, Wen Y, Tian Z et al. A one-pot isothermal Cas12-based assay for the sensitive detection of microRNAs. *Nat Biomed Eng* 2023;**7:**1583–601. https://doi.org/10.1038/s41551-023-01033-1

[4] Malcı K, Walls LE, Rios-Solis L. Rational design of CRISPR/Cas12a-RPA based one-pot COVID-19 detection with design of experiments. *ACS Synth Biol* 2022;**11:**1555–67. https://doi.org/10.1021/acssynbio.1c00617

[5] Hu M, Qiu Z, Bi Z et al. Photocontrolled crRNA activation enables robust CRISPR-Cas12a diagnostics. *Proc Natl Acad Sci USA* 2022;**119:**e2202034119. https://doi.org/10.1073/pnas.2202034119

[6] Chen Y, Wang X, Zhang J et al. Photoactivatable CRISPR/Cas12a strategy for one-pot DETECTR molecular diagnosis. *Anal Chem* 2022;**94:**9724–31. https://doi.org/10.1021/acs.analchem.2c01146

[7] Lin M, Yue H, Tian T et al. Glycerol additive boosts 100-fold sensitivity enhancement for one-pot RPA-CRISPR/Cas12a assay. *Anal Chem* 2022;**94:**8277–84. https://doi.org/10.1021/acs.analchem.2c00616

[8] de Puig H, Lee RA, Najjar D et al. Minimally instrumented SHERLOCK (miSHERLOCK) for CRISPR-based point-of-care diagnosis of SARS-CoV-2 and emerging variants. *Sci Adv* 2021;**7:**eabh2944. https://doi.org/10.1126/sciadv.abh2944

[9] Tong X, Zhang K, Han Y et al. Fast and sensitive CRISPR detection by minimized interference of target amplification. *Nat Chem Biol* 2024;20:885–93. https://doi.org/10.1038/s41589-023-01534-9

[10] Cao Y, Lu X, Lin H et al. CoLAMP: CRISPR-based one-pot loop-mediated isothermal amplification enables at-home diagnosis of SARS-CoV-2 RNA with nearly eliminated contamination utilizing amplicons depletion strategy. *Biosens Bioelectron* 2023;**236:**115402. https://doi.org/10.1016/j.bios.2023.115402

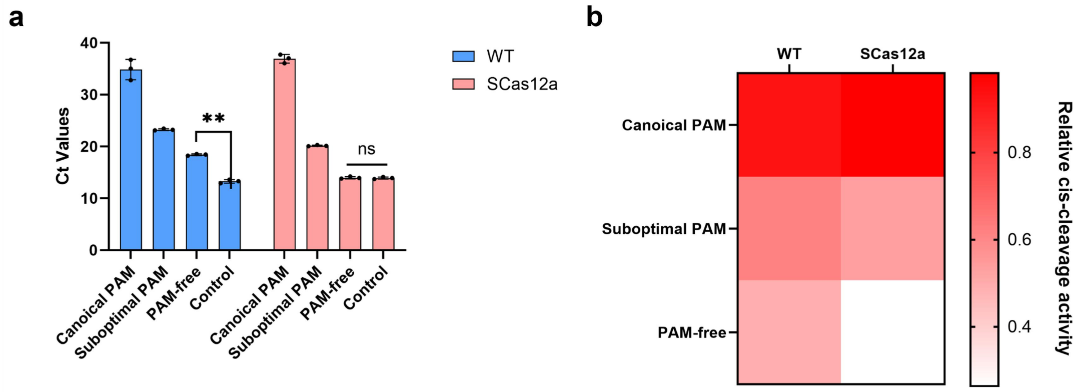

Fig. S1. qPCR-based assessment of *cis*-cleavage activity in wild-type Cas12a and SCas12a. a, dsDNA cleavage across PAM contexts: canonical , suboptimal, and PAM-free. Samples containing identical dsDNA concentration but lacking Cas12a RNP served as controls Statistical significance was assessed using a two-tailed unpaired t-test: ns, $p > 0.05$; *, $p \leq 0.05$; **, $p \leq 0.01$; ***, $p \leq 0.001$; ****, $p \leq 0.0001$. **b,** WT Cas12a versus SCas12a indicated by heatmap; darker shading indicates lower residual dsDNA signal—and thus higher *cis*-cleavage activity.

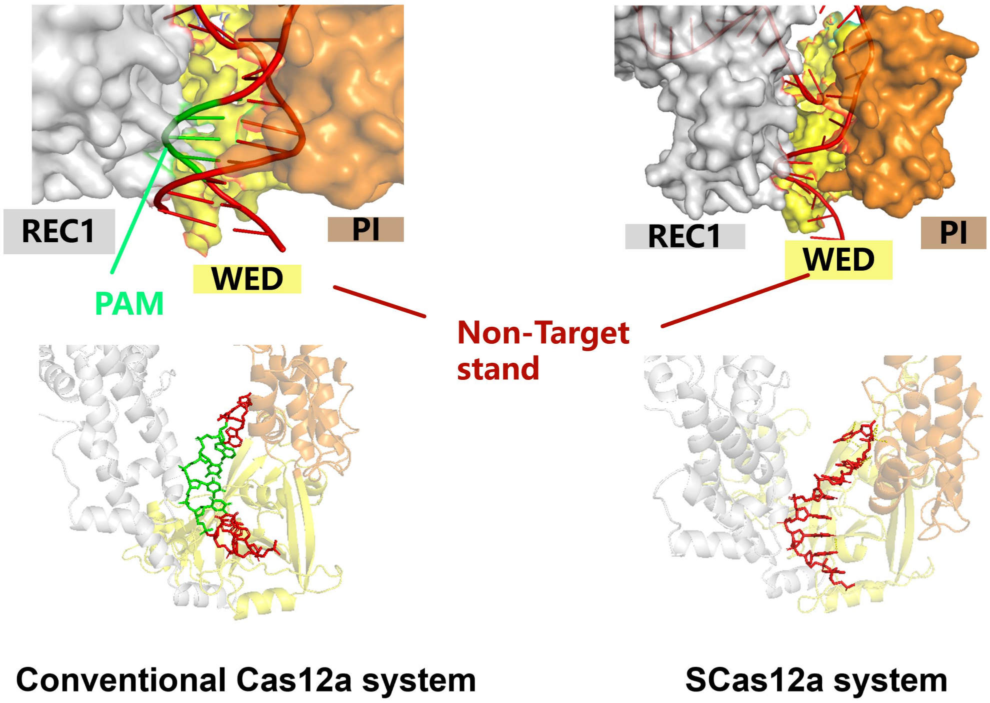

Fig. S2. Comparison of the active centers between the conventional Cas12a and SCas12a systems. Structural analysis indicated that even in the absence of a PAM sequence within the NTS of dsDNA, unwinding can still occur, albeit to a lesser extent. Moreover, longer DNA chains are associated with greater difficulty in unwinding and detection.

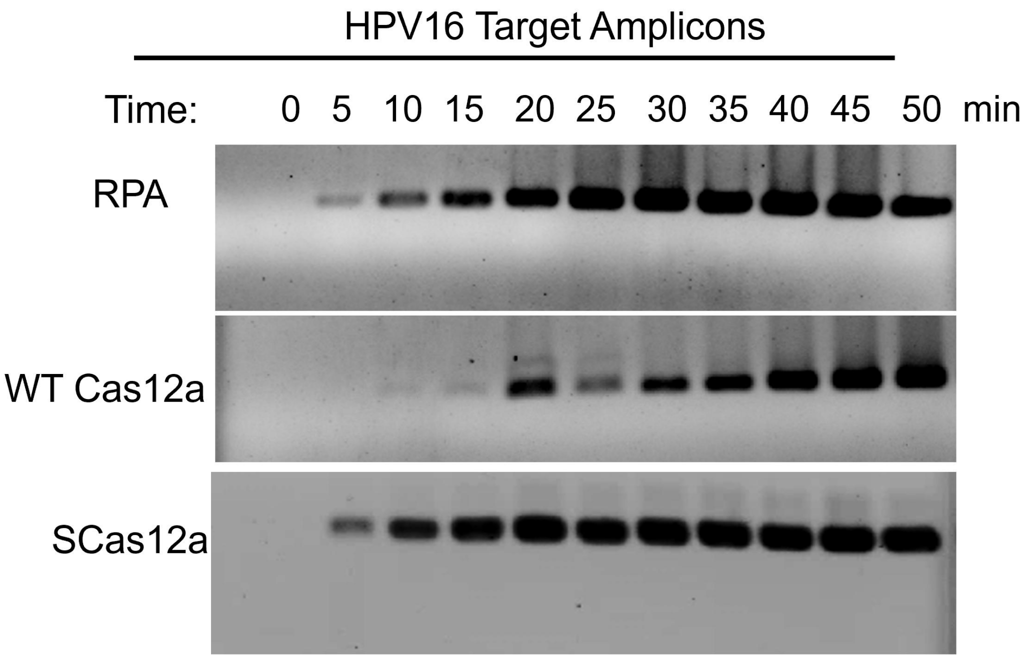

Fig. S3. Comparison of amplicon accumulation across one-pot reactions containing (i) RPA alone, (ii) RPA supplemented with wild-type Cas12a RNP, or (iii) RPA supplemented with SCas12a RNP. Reactions were assembled with 100 nM Cas12a RNP and 1 pM dsDNA substrate, incubated at 37°C, and sampled at 0, 5, 10, 15, 20, 25, 30, 35, 40, 45, and 50 minutes. Amplicons were resolved by agarose gel electrophoresis.

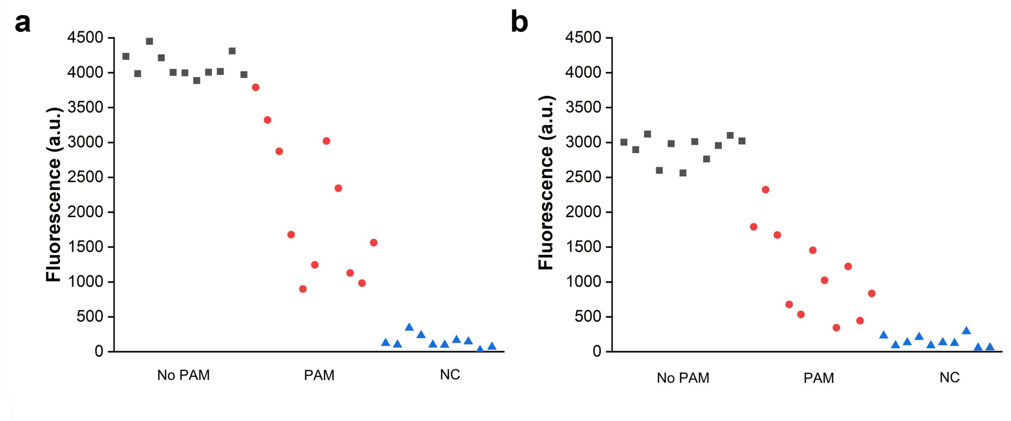

Fig. S4. The reliability of SCas12a-mediated one-pot reactions. The reliability of the one-pot assay was evaluated using either PAM-free or canonical PAM targeting (a) the *L1* gene of HPV16 and (b) the *Orf1ab* gene of SARS-CoV-2.

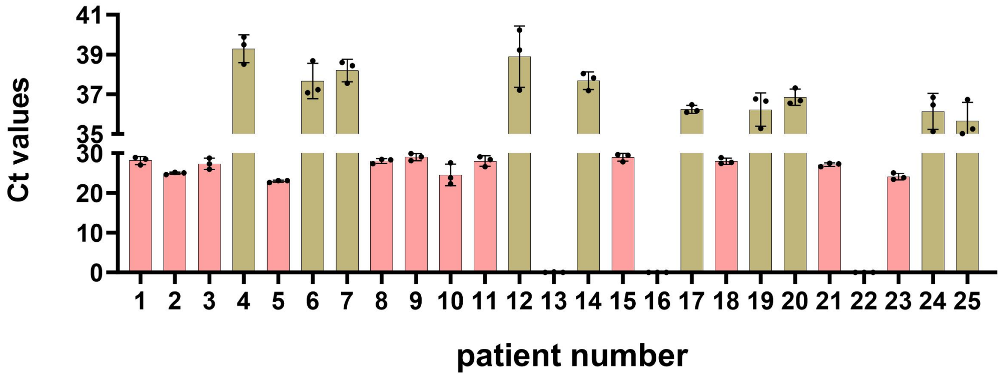

Fig. S5. Indentification of HPV16 in 25 human vaginal secretion samples by qPCR. The pink color with a Ct value below 35 indicates positive results. The absence of a Ct value or the presence of olive green color with a Ct value above 35 indicates negative results. Error bars represent the mean value ± standard deviation from three technical replicates.

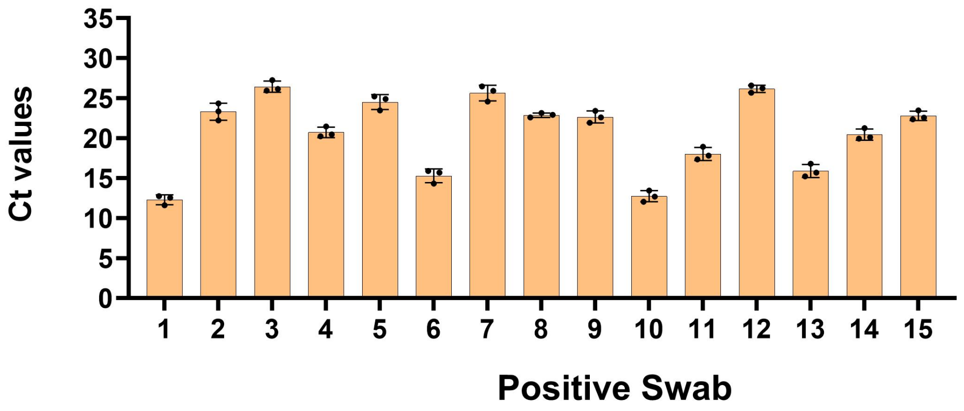

Fig. S6. Identification of 15 positive SARS-CoV-2 infected patients from nasopharyngeal swabs using RT-qPCR. The brown color with a Ct value below 35 indicates positive results. Error bars represent the mean value ± standard deviation from three technical replicates.

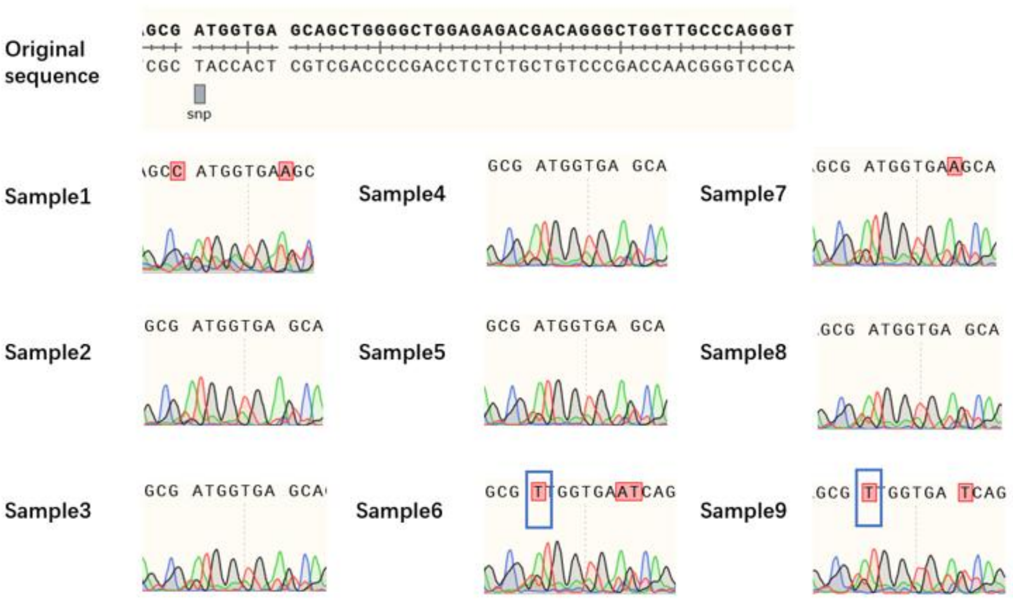

Fig. S7. Sanger sequencing of the TP53 (rs2151029805) from nine OSCC tissues. The sequencing data revealed that samples 6 and 9 harbor A>T mutations.
